# Connexin 43 in Pathophysiology of Cardiac Diseases: From Molecular Mechanisms to Therapeutic Strategies

**DOI:** 10.33549/physiolres.935633

**Published:** 2025-12-01

**Authors:** Lucia ŽIGOVÁ, Orsolya HRUBÁ, Jan KYSELOVIC, Andrea GAŽOVÁ

**Affiliations:** 1 Institute of Pharmacology and Clinical Pharmacology, Faculty of Medicine, Comenius University Bratislava, Bratislava, Slovakia; 2 5th Department of Internal Medicine, Faculty of Medicine, Comenius University Bratislava, Bratislava, Slovakia; 3 Department of Pharmacology and Toxicology, University of Veterinary Medicine and Pharmacy, Košice, Slovakia

**Keywords:** Connexin 43, Gap Junctions, Myocardial dysfunction, Peptidomimetics

## Abstract

Connexin 43 (Cx43) plays a vital role in maintaining myocardial function through gap junctions (GJs) and hemichannels (HCs), facilitating crucial intercellular communication and ion exchange. Its regulation is precisely controlled by various signaling pathways that influence its phosphorylation status, trafficking, and degradation, thereby modulating myocardial function under physiological and pathological conditions. Under pathological conditions such as ischemic injury, cardiomyopathies, or heart failure, Cx43 undergoes dephosphorylation and is mislocalized from GJs at intercalated discs to the lateral membrane. This disruption in intercellular connectivity impairs electrical conduction and increases susceptibility to arrhythmias, with the loss of functional Cx43-mediated GJs further exacerbating myocardial dysfunction and contributing to disease progression. Given the critical role of Cx43 in cardiac pathology, therapeutic strategies targeting Cx43, particularly peptidomimetics, have emerged as promising cardioprotective approaches. These small synthetic peptides selectively modulate Cx43 HC activity, preventing excessive cellular stress and preserving intercellular communication. Recent advancements, including TAT-conjugated peptides and Hdc-modified analogues, have enhanced the efficacy of peptidomimetics by improving cellular uptake and therapeutic effectiveness. This review highlights the role of Cx43 and Cx43-derived peptidomimetics in cardiovascular diseases, noting their promising potential for broader clinical applications due to Cx43 dysregulation being implicated in various pathologies.

## Introduction

Cardiovascular diseases are among the leading causes of morbidity globally, placing significant pressure on healthcare systems and impacting patient quality of life [1]. Among these, myocardial disorders represent a critical subset characterized by progressive loss in the structural and functional integrity of the heart muscle. Various pathological stimuli, including ischemic injury, pressure overload or genetic predispositions, can deform the standard myocardial structure and contribute to the progression of cardiac dysfunction [2,3].

A key factor in the pathophysiology of these disorders is the impaired intercellular communication within the myocardium, mediated by specialized cell-cell junctions known as gap junctions (GJs). The major protein component of these junctions in the heart is connexin 43, which facilitates the efficient transfer of ions and small molecules between adjacent cardiomyocytes, enabling electrical conduction and supporting metabolic activity. Cx43 downregulation and redistribution have been identified as critical events leading to cardiac arrhythmias, impaired myocardial contractility, and the progression of heart failure [4–7].

Given its critical role in maintaining myocardial function, connexin 43 has emerged as a promising molecular target for developing novel therapeutic strategies. Mimetic peptides, small synthetic molecules designed to replicate specific functional domains of Cx43 and selectively modulate its activity, represent a promising approach for preserving myocardial integrity and function.

## Function of Gap Junctions in the Myocardium

Electrical conductivity in the myocardium is enabled by gap junctions (GJ), which link cells *via* specialized proteins known as connexins. These proteins form the subunits of hemichannels, enabling efficient signal transmission between cells [8]. While connexins play a critical role in the heart, they are not limited to cardiac tissue. Connexin isoforms are widely expressed in various organs and systems such as the brain (e.g., Cx26 in neurons, Cx43 in astrocytes) [9], liver (Cx32 in hepatocytes) [10], retina (Cx35 and Cx36) [11] or vascular system (Cx37 and Cx40) [12].

GJ channels are essential for electrical communication among cardiomyocytes and the coordination of their contraction. Disruption of GJ-mediated signaling can lead to arrhythmia or impaired contractile function of the myocardium. Although some mechanisms underlying GJ remodeling have been identified, the precise pathways by which it contributes to the development of human heart failure remain unclear [8].

GJs in cardiac tissue mediate intercellular communication, thereby maintaining normal heart rhythm, regulating vascular tone, and facilitating metabolic exchange. They enable the transfer of small molecules less than 1 kDa, such as ATP, cAMP, IP3, and glucose involved in cell growth, differentiation, and function [13–15]. GJ connectivity in excitable cells such as cardiomyocytes is essential for mediating electrical coupling, action potential propagation, and ensuring synchronous myocardial contraction. Importantly, GJs are crucial not only for cardiomyocytes but also for smooth muscle and endothelial cells, contributing to the integrity of the entire cardiovascular system [16].

## Characteristics of Gap Junctions

The name “connexin” originates from its function in facilitating intercellular connections [17]. Studies involving the isolation and sequencing of Cx43 from tissues such as the heart and liver have demonstrated its essential role in forming GJs channels with a unique structural composition [18].

A connexon is a hemichannel (HC) composed of six connexin subunits. The association of two hemichannels from adjacent cells forms a dodecameric gap junction channel that allows direct intercellular communication.

Cx43 HCs are found predominantly at the lateral membrane of cardiomyocytes and are largely closed in physiological settings. Under pathophysiological conditions they open, disturbing ionic homeostasis and promoting proinflammatory and profibrotic processes. This may contribute pathological conditions, such as cardiac arrhytmias, neurodegenerative and chronic inflammatory diseases [19,20].

**Fig. 1:**
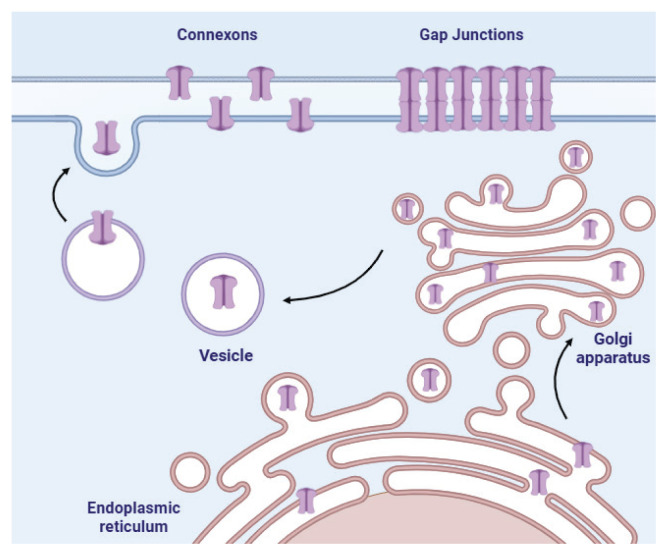
Biosynthesis and transport of hemichannels in the cell. Before their assembly into GJs, HCs remain closed, thereby preventing the uncontrolled passage of molecules and ions. While GJs typically close under certain pathological conditions, HCs exhibit the opposite response by opening during pathological stimuli [24]. Created with BioRender.com.

Connexins are characterized by four transmembrane domains, two extracellular loops, one cytoplasmic loop, and variable N- and C-termini. The C-terminal domain is the most variable region and determines many isoform-specific regulatory features, including interactions with intracellular signaling proteins [21]. Connexins are synthesized at membranes of the rough endoplasmic reticulum and transported through the Golgi apparatus to the plasma membrane. Upon reaching the plasma membrane, the connexons formed by connexins align with those on adjacent cells, thereby giving rise to functional GJs [22]. This complex process is tightly regulated to ensure GJ’s proper localization and functionality across various tissues (Fig. 1).

**Fig. 2:**
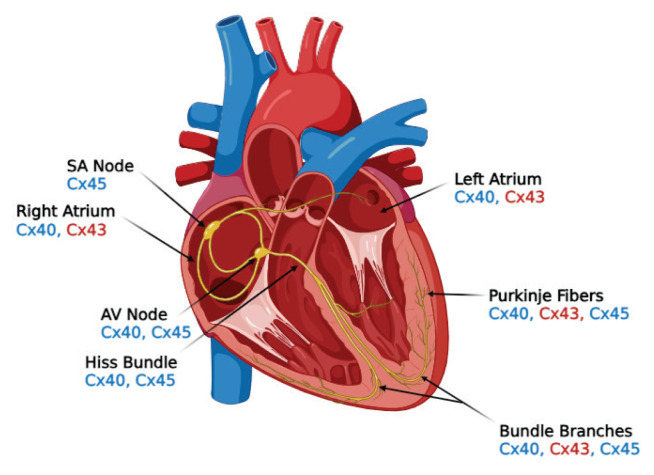
Localization of connexins in the myocardium

Twenty-one genes encoding connexins have been identified, and more than 12 phosphorylation sites of Cx43 have been reported. The proteins encoded by these genes are classified according to their molecular weights, which range from 26 to 60 kDa [23]. Three main connexin isoforms in the adult human heart have been described - named based on their predicted molecular weights: Cx40, Cx43, and Cx45 (Fig. 2). Cx43, encoded by the *GJA1* gene, is the predominant isoform in working cardiomyocytes and plays a key role in maintaining the electrical stability of the myocardium [25]. The role of Cx43 in cardiac electrophysiology has been demonstrated in various knockout models. Germline deletion of *Gja1* (Cx43^−^/^−^) in mice results in postnatal death [26]. In contrast, cardiomyocyte-specific deletion of Cx43 in mice leads to progressive conduction slowing and sudden arrhythmic death by two months of age, despite the absence of overt structural abnormalities [27]. Although other connexins are co-expressed in the heart, they appear insufficient to fully compensate for the loss of Cx43.

It is important to note that the dose-response relationship between Cx43 and phenotype is not linear. In a rat volume overload model, the minimum conduction velocity was increased in mildly hypertrophied hearts despite a decrease in total and phosphorylated Cx43, whereas a significant slowing of conduction was observed in the most severely hypertrophied hearts. Both total and phosphorylated Cx43 decreased proportionally with the severity of the phenotype [28].

**Fig. 3:**
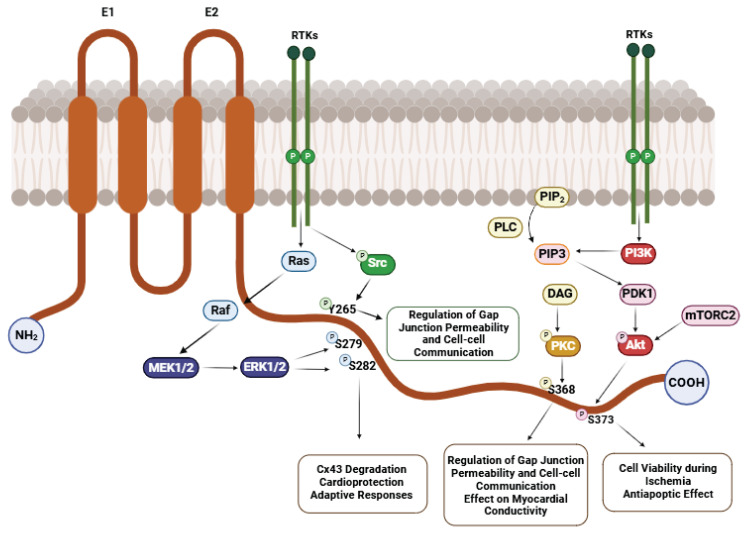
Cx43 Structure and Signaling Cascades. The most prominent phosphorylation sites are on the C-terminal end of connexin 43. The figure shows tyrosine and serine residues regulated by the signaling cascades Src, MAPK/ERK, PKC, and PI3K/Akt. Created with BioRender.com.

Despite exhibiting distinct biophysical properties, This domain contains multiple serine, threonine, and tyrosine phosphorylation sites, thereby regulating connexin function [29]. Both the amino and carboxy-terminal ends of Cx43 are located in the cytoplasm and encompass four hydrophobic transmembrane α-helical domains linked by two extracellular loops (E1 and E2) and a single cytoplasmic loop (CL) (Fig. 3). The Cx43 CT mediates interactions with proteins such as the tight junction protein Zonula Occludens-1 (ZO-1) [30], which regulates GJ size [31,32]. In ventricular cardiomyocytes, ZO-1 is primarily localized at the intercalated discs, forming functional interactions with GJ containing Cx43 and regulating their size and stability. These interactions are essential for maintaining proper GJ organization and ensuring efficient signal transmission [4].

Alterations in Cx43 expression have been observed in various diseases, including hypertrophic cardiomyopathy, ischemia, and fibrosis. Redistribution of Cx43 can lead to arrhythmogenesis, particularly following ischemia-reperfusion injury (I/R). Recent studies have demonstrated that mutations or downregulation of these proteins can significantly reduce Cx43 levels and impair GJ functionality, resulting in severe disruption of electrical impulse propagation in cardiac tissue [32–34].

Recent studies indicate that Cx43 also plays a role in mitochondria, enhancing cell survival and protecting against apoptosis. This protective function is particularly significant under stress conditions such as ischemia or myocardial infarction. Dephosphorylation of Cx43 can be associated with dysfunction of this protein and contribute to ventricular arrhythmias [33]. Upregu-lation of mitochondrial Cx43 may exert a protective effect following myocardial infarction by reducing ischemic injury and cardiomyocyte apoptosis [35].

## Cx43 Signaling Pathways

Regulation of GJs involves several post-translational modifications, including phosphorylation [24], ubiquitylation [36], and SUMOylation [37]. In addition to these modifications, connexin 43 (Cx43) expression is also controlled at the post-transcriptional level by microRNA-1 [38].

Below, we summarize key signaling pathways that modulate gap junctions primarily through phosphorylation-dependent mechanisms. These include mitogen-activated protein kinase (MAPK), protein kinase C (PKC), phosphoinositide 3-kinase/Akt (PI3K/Akt), and Src kinase pathways - all of which significantly influence Cx43 function and localization in the context of cardiovascular diseases (Fig. 3). These signaling pathways modulate essential cardiac functions such as cell proliferation, apoptosis, inflammation, and electrical conductivity [39]. Given that Cx43 is the principal component of GJ channels in cardiac tissue, its precise regulation through these pathways is critical for maintaining cardiac homeostasis and function.

## MAPK Signaling Pathway

**Fig. 4:**
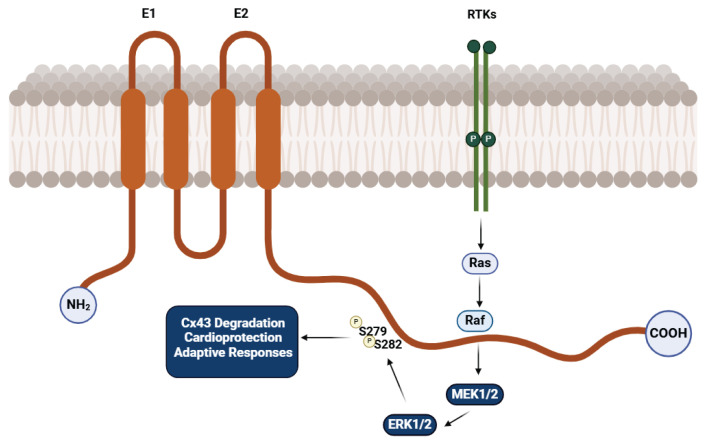
Role of the MAPK Signaling Pathway in Connexin 43 Regulation. Created with BioRender.com.

The MAPK signaling pathway is critical in regulating Cx43, influencing its expression, phosphorylation, and cardiomyocyte function. Studies have shown that hypoxia induces Cx43 dysregulation by activating MAPK pathways, particularly MEK1/2 and ERK1/2, resulting in reduced Cx43 levels and impaired myocardial electrical conductivity [40] (Fig. 4).

Phosphorylation of Cx43 through the MAPK signaling pathway, particularly at sites S255, S262, S279, and S282, has been shown to promote the binding of cyclin E, leading to increased proliferation of smooth muscle cells. The mechanisms regulating Cx43 via the MAPK signaling pathway include phosphorylation, which influences the functional properties and localization of Cx43 within the plasma membrane [41,42].

## PKC Signaling Pathway

**Fig. 5:**
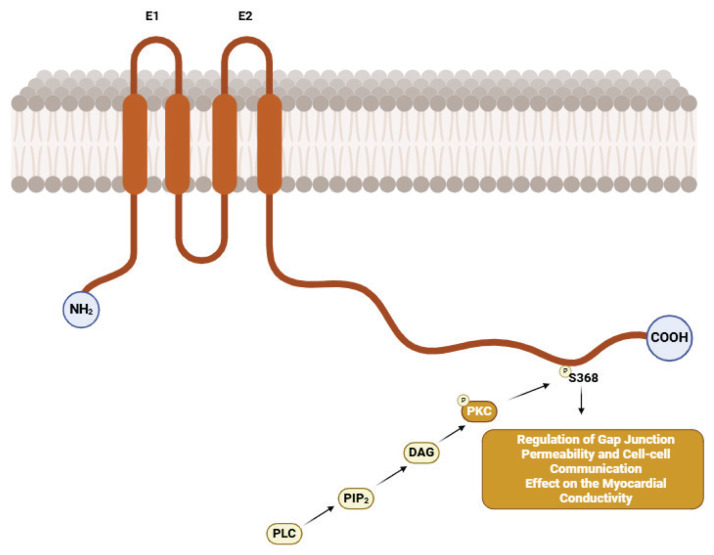
Role of the PKC Signaling Pathway in Connexin 43 Regulation. Created with BioRender.com.

Protein kinase C (PKC) is a crucial regulatory mechanism for Cx43 and is pivotal in controlling cardiomyocyte GJ. PKC is activated by secondary messengers such as diacylglycerol (DAG), which are released in response to various stimuli, including stress (Fig. 5) [7,43].

Phosphorylation of Cx43 by PKC can alter its intracellular localization and functional activity. Upon activation, PKC promotes the internalization of Cx43 from the plasma membrane, directing it toward degradation through the lysosomal pathway. This regulation can significantly impact myocardial electrical conductivity and contribute to the development of arrhythmias. PKC signaling regulates Cx43 by modulating the stability, facilitating interactions, promoting the internalization into intracellular compartments, and promoting lysosomal degradation, ultimately reducing GJs [7,43].

## PI3K/Akt Signaling Pathway

**Fig. 6:**
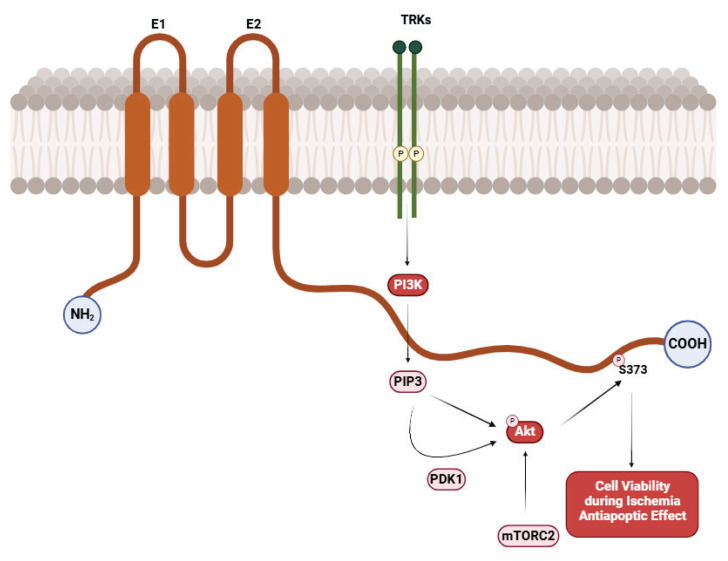
Role of the PI3K/Akt Signaling Pathway in Connexin 43 Regulation. Created with BioRender.com.

Protein kinase B (Akt) is a critical regulator of cardiomyocytes’ growth, cell survival, and metabolism. The PI3K/Akt signaling pathway activates *via* phosphatidylinositol-3-kinase (PI3K) in response to various extracellular stimuli, such as growth factors or insulin. Phosphorylation of Cx43 by Akt can influence its stability and functionality, while Akt activation contributes to enhanced cellular resistance to apoptosis and supports myocardial repair. The PI3K/Akt signaling pathway is crucial in adapting cardiac tissue to stress factors, such as ischemia. Mechanisms regulating Cx43 through the Akt signaling pathway include phosphorylation, which impacts cell survival, modulates interactions with other proteins, and provides protective effects against apoptosis via Akt activation [44,45] (Fig. 6).

## Src Signaling Pathway

Activation of the Src signaling pathway is regulated by binding growth factors or integrins to their receptors, leading to the phosphorylation of Src at activation sites. Phosphorylation of Cx43 by Src induces internalization and degradation, thereby reducing the number of functional GJs in the plasma membrane. The Src signaling pathway can impair intercellular communication, disrupt myocardial conductivity, and contribute to arrhythmogenic conditions [24,46].

## Role of Connexin 43 in Ischemic Cardiac Injury and Heart Failure

Signaling pathways modulate the phosphorylation, localization, and stability of Cx43. Dysregulation of intercellular signaling mechanisms disrupts cellular communication, leading to impaired GJ function and contributing to the progression of cardiovascular diseases [4].

During cardiac ischemic injury, Cx43 undergoes dephosphorylation and redistribution, leading to the displacement of GJs from intercalated discs to the lateral membranes of cardiomyocytes. This disruption in Cx43 phosphorylation and localization impairs intercellular electrical coupling, increasing the risk of arrhythmias. Additionally, unopposed Cx43HCs can open under ischemic conditions, disrupting cellular ionic homeostasis and leading to further myocardial damage [29,47].

Similar mechanisms involving altered phosphorylation and localization of Cx43 also play a crucial role in the pathogenesis of heart failure. Disrupted intercellular communication impairs integrity, slows electrical conduction, and elevates the risk of arrhythmias [48]. These processes correlate with increased interaction between Cx43 and the scaffolding protein ZO-1, resulting in reduced GJ size and further deterioration of electrical and mechanical myocardial stability [4,49].

## Role of Connexin 43 in Cardiac Hypertrophy and Cardiomyopathies

Cardiac hypertrophy induced by the increased mechanical load is associated with significant remodeling of cardiac tissue, characterized by altered Cx43 phosphorylation, expression, and localization. Mechanical stress leads to reduced phosphorylation of Cx43 at S368, causing decreased expression of Cx43. Under these conditions, Cx43 undergoes lateral redistribution from intercalated discs to lateral cell membranes - a pattern similar to changes observed during ischemic injury [50,51]. The hypertrophic remodeling of GJs occurs progressively, with early stages characterized by regular or mildly elevated Cx43 expression, whereas in advanced stages, expression of Cx43 significantly declines, accompanied by heterogeneous distribution of Cx43 [49,51,52].

Similar mechanisms involving abnormal phosphorylation, function and distribution of Cx43 have also been observed in the hearts of patients with Duchenne muscular dystrophy (DMD), an inherited neuro-degenerative disorder affecting cardiac and skeletal muscle caused by mutations in the dystrophin gene. Although DMD is primarily recognized as a neuromas-cular disorder rather than a cardiovascular disease, the development of dilated cardiomyopathy represents a significant pathological consequence. It remains the leading cause of mortality in affected individuals. Loss of proper post-translational phosphorylation of Cx43 at residues S325/S328/S330 further contributes to impaired GJ communication, exacerbating electrical instability, arrhythmogenic potential, and progression toward heart failure [53,54].

Given the essential role of Cx43 in maintaining cardiac homeostasis, research is increasingly focused on therapeutic strategies aimed at its targeted modulation. Peptidomimetics represents a promising therapeutic approach, offering selective regulation of Cx43 function while supporting restoring its physiological regulation.

## Peptidomimetics Targeting Connexin 43: An Emerging Therapeutic Perspective

Various therapeutic strategies, including monoclonal antibodies, antisense oligonucleotides, and synthetic molecules, can mediate the modulation of Cx43. However, Cx43 therapeutic modulation requires careful consideration of tissue- and compartment-specific expression levels. Excessive or untargeted upregulation may lead to pathological changes such as lateralization or proarrhythmic remodeling [55].

Peptidomimetics represents a class of synthetic peptides that mimic short sequences of Cx43, enabling targeted modulation of its function (Table 1). These peptides selectively interact with pathologically activated HCs, thereby minimizing adverse effects on physiological intercellular communication. Unlike non-mimetic peptides, peptidomimetics offers higher affinity and specificity toward target protein domains. Their mechanism of action relies on recognizing native sequences within the Cx43 structure, allowing precise intervention in its regulatory mechanisms [22,56,57].

## Gap19

The mimetic nonapeptide Gap19, a selective inhibitor of Cx43 hemichannels, binds to the CT domain of Cx43, preventing the CT-CL interaction and thereby inhibiting HC activation during ischemia. Gap19 specifically blocks Cx43 HCs without affecting GJs. Instead of directly occluding the HC pore, Gap19 increases the membrane voltage threshold required for activation and selectively restricts permeability to low-molecular-weight compounds. The amino acid residue I130 is thought to be critical in this interaction, as it contributes to the formation of hydrogen bonds that is likely essential for the inhibitory efficacy of Gap19 [23,59,60].

Studies show that inhibition of Cx43 HCs using Gap19 alleviates I/R injury in the myocardium. Significant cardiomyocyte death was observed in in vitro models of OGD/acidosis, whereas applying Gap19 before OGD/acidosis improved cell viability by approximately 30 % and reduced cellular oedema. Similarly, in an in vivo mouse model of myocardial I/R injury, intravenous administration of Gap19 before ischemia reduced infarct size by 20 % compared to controls [59].

To enhance the efficacy of Gap19, a modified version, TAT-Gap19 was developed by conjugating Gap19 with the TAT sequence derived from the HIV Tat protein. The TAT internalization sequence improves membrane permeability, facilitating more efficient cellular uptake of the peptide and enhancing its ability to reduce cellular stress and inflammation. TAT-Gap19 has been shown to suppress arrhythmogenic membrane depolarizations, such as delayed afterdepolarizations under adrenergic stimulation in heart failure models [61]. The application of TAT-Gap19 achieves a half-maximal inhibition of Cx43 hemichannels at a fivefold lower concentration compared to conventional Gap19, making it a more potent and effective therapeutic option [62,63].

## Gap26, Gap27

Peptide mimetics Gap26 and Gap27 are derived from the extracellular loop domains of Cx43, with Gap26 originating from the extracellular loop E1 and Gap27 from the extracellular loop E2 [24]. Research suggests that these peptides primarily target HCs rather than GJ channels, and the likely mechanism of action involves direct interaction with HCs, leading to their rapid inhibition within minutes. In contrast, GJ channel inhibition occurs over a longer time frame [64].

Studies on isolated rat cardiomyocytes suggest that Gap26 protects against ischemic injury. Its application before or during simulated I/R significantly enhanced cell survival compared to the control peptide sGap26, which did not exhibit a protective effect. Moreover, Gap26 preserved mitochondrial activity at a higher level, whereas untreated cells experienced a significant decline. Further experiments demonstrated that Gap26 reduced Cx43 HC activity by approximately 60 %, whereas the control peptide sGap26 had no effect [65].

A recent advancement in the innovation of the mimetic peptide Gap27 is the development of a lipidated version of a Gap27-like peptide, achieved by adding a hexadecyl lipid group (SRPTEKT-Hdc), which exhibits enhanced inhibitory efficacy compared to its non-lipidated form. Lipidation of the SRPTEKT sequence increased the peptide’s ability to inhibit both HCs and GJs mediated by Cx43, with significantly higher efficacy observed for the phosphorylated form of Cx43 (pS368) compared to its dephosphorylated counterpart. These findings suggest SRPTEKT-Hdc interacts with Cx43 channels conformation-specific, dependent on phosphorylation at S368 [66].

## αCT1

The αCT1 peptidomimetic is derived from the last nine amino acids of the CT sequence of the Cx43 protein. Its primary mechanism of action involves specific binding to the PDZ2 domain of ZO-1. The interaction between the PDZ2 domain of ZO-1 and Cx43 is crucial in regulating GJ assembly and CxHC activity. By disrupting this interaction, αCT1 alters cellular junction dynamics, leading to increased formation of GJ plaques, which enhances intercellular communication, while simultaneously reducing Cx43 HC activity, which has been implicated in various pathological conditions [60,67].

Administration of αCT1 following ischemic injury contributed to preserving GJ structure and function within the intercalated discs. This treatment enhanced Cx43 presence in ischemically affected regions. It promoted stable distribution, which was linked to decreased incidence and severity of induced arrhythmias and faster myocardial depolarization. Furthermore, αCT1 treatment enhanced the phosphorylation of Cx43 at S368 via PKC-ɛ, representing a key regulatory mechanism of the Cx43 function. This effect was inhibited in the presence of PDZ2, highlighting the complex regulatory role of ZO-1 in the mechanism of action. As a result of these modifications, a significant improvement in left ventricular contractile function was observed during reperfusion. Hearts treated with αCT1 exhibited better systolic and diastolic function compared to control groups, correlating with increased Cx43-pS368 phosphorylation and enhanced stabilization of GJs at intercalated discs [67,68].

The therapeutic benefits of αCT1 have been recognized across multiple medical disciplines. Preclinical research has demonstrated that αCT1 promotes faster wound healing while minimizing inflammation and scarring. Its application is also being investigated in diabetic complications, radiation-induced skin damage, and radiation dermatitis [58,67]. Additionally, promising results have been observed in oncology, where αCT1 stabilized Cx43 localization at GJs in breast cancer cells, enhancing GJ activity while impairing tumor cell proliferation and survival [56].

## Conclusion

Connexin 43 is crucial in maintaining myocardial function in gap junctions and hemichannels, facilitating intercellular communication and ion exchange. The regulation of connexin 43 is tightly controlled by various signaling cascades, which influence the phosphorylation status, trafficking, and degradation, thereby modulating myocardial function under physiological and pathological conditions. While gap junctions ensure synchronized electrical conduction between cardiomyocytes, hemichannels contribute to extracellular signaling and cellular homeostasis. Cx43 undergoes pathological dephosphorylation and is mislocalized from gap junctions at intercalated discs to the lateral membrane in conditions such as ischemic injury, cardiomyopathies or heart failure. This disrupts intercellular connectivity, impairing electrical conduction and increasing susceptibility to arrhythmias. The loss of functional connexin 43-mediated gap junctions further exacerbates myocardial dysfunction, contributing to disease progression.

Given the critical role of Cx43 in cardiac pathology, therapies targeting Cx43 - particularly peptidomimetics - have emerged as promising cardioprotective strategies. These small synthetic peptides selectively modulate Cx43 hemichannel activity, preventing excessive cellular stress and restoring intercellular communication. Notably, recent advancements, such as TAT-conjugated peptides and Hdc-modified analogues, have enhanced the efficacy of peptidomimetics by improving cellular uptake and therapeutic effectiveness.

This review article focuses on the role of Cx43 and Cx43-derived peptidomimetics in treating cardiovascular diseases. However, their therapeutic potential extends beyond this field. Given that Cx43 dysregulation is implicated in the pathogenesis of various diseases, targeted modulation of Cx43 function *via* peptidomimetics remains an active area of research and holds promise for broader clinical applications.

## Figures and Tables

**Table 1:** Characterization of Mimetic Peptides Targeting Connexin43 [22,58]

Peptide name	Amino acid residues	Amino acid sequence	cx43 domain localization
*GAP19*	128–136	KQIEIKKFK	Intracellular
*TAT-GAP19*	TAT 128–136	YGRKKRRQRRRKQIEIKKFK	Intracellular
*GAP26*	64–76	VCYDKSFPISHVR	Extracellular
*GAP27*	201–211	SRPTEKTIFII	Extracellular
*GAP27-HDC*	201–207-Hexadecyl Lipid Tail	SRPTEKT-Hdc	Extracellular
*ACT1*	Antennapedia 374–382	RQPKIWFPNRRKPWKKRPRPDDLEI	Intracellular

## Abbreviations

Akt: Protein Kinase B
ATP: Adenosine Triphosphate
AV Node: Atrioventricular Node
cAMP: Cyclic Adenosine Monophosphate
CL: Cytoplasmatic Loop
CT: Carboxyterminal Domain
Cx43: Connexin 43
DAG: Diacylglycerol, DMD, Duchenne Muscular Dystrophy
ERK1/2: Extracellular signal-regulated Kinases 1 and 2
GJ: Gap Junction
HCs: Hemichannels
IP3: Inositol Triphosphate
I/R: Ischemia/Reperfusion
MAPK: Mitogen-Activated Protein Kinase
MEK1/2: Mitogen Activated Kinases 1 and 2
mTORC2: Mammalian target of Rapamycin C2
OGD: Oxygen Glucose Deprivation
PDK1: Phosphoinositide-dependent Kinase-1
PI3K: Phosphoinositide 3-Kinase
PIP2: PIP2-Phosphatidylinositol-4,5-bisphosphate
PKC: Protein Kinase C
PLC: Phospholipase C
RTK: Receptor Tyrosine Kinase
SA Node: Sinoatrial Node
TAT: Trans-activator of Transcription
ZO-1: Zonula Occludens-1
